# Regulation and Directing Stem Cell Fate by Tissue Engineering Functional Microenvironments: Scaffold Physical and Chemical Cues

**DOI:** 10.1155/2019/2180925

**Published:** 2019-12-27

**Authors:** Fei Xing, Lang Li, Changchun Zhou, Cheng Long, Lina Wu, Haoyuan Lei, Qingquan Kong, Yujiang Fan, Zhou Xiang, Xingdong Zhang

**Affiliations:** ^1^Department of Orthopaedics, West China Hospital, Sichuan University, No. 37 Guoxue Lane, Chengdu, 610041 Sichuan, China; ^2^Department of Pediatric Surgery, West China Hospital, Sichuan University, No. 37 Guoxue Lane, Chengdu, 610041 Sichuan, China; ^3^National Engineering Research Center for Biomaterials, Sichuan University, 610064 Chengdu, Sichuan, China

## Abstract

It is well known that stem cells reside within tissue engineering functional microenvironments that physically localize them and direct their stem cell fate. Recent efforts in the development of more complex and engineered scaffold technologies, together with new understanding of stem cell behavior in vitro, have provided a new impetus to study regulation and directing stem cell fate. A variety of tissue engineering technologies have been developed to regulate the fate of stem cells. Traditional methods to change the fate of stem cells are adding growth factors or some signaling pathways. In recent years, many studies have revealed that the geometrical microenvironment played an essential role in regulating the fate of stem cells, and the physical factors of scaffolds including mechanical properties, pore sizes, porosity, surface stiffness, three-dimensional structures, and mechanical stimulation may affect the fate of stem cells. Chemical factors such as cell-adhesive ligands and exogenous growth factors would also regulate the fate of stem cells. Understanding how these physical and chemical cues affect the fate of stem cells is essential for building more complex and controlled scaffolds for directing stem cell fate.

## 1. Introduction

Stem cells have the ability of self-renewal and differentiation; they can be used to repair the bone, cartilage, and skin and play an important role in regenerative medicine [1, 2]. Stem cells are generally classified into embryonic stem cells and adult stem cells. Embryonic stem cells are more primitive, but some studies have shown that they may turn into tumor cells, which dramatically limits their application. At present, adult stem cells, such as bone marrow-derived mesenchymal stem cells (BMMSCs), adipose-derived stromal cells (ASCs), umbilical cord-derived mesenchymal stem cells (UC-MSCs), and even urine-derived mesenchymal stem cells (U-MSCs), have attracted more and more attention and are widely used in the field of regenerative medicine [3]. In the field of tissue engineering regeneration, regulating the proliferation and differentiation of stem cells has been an important research direction for stem cells [4, 5].

The fate of stem cells includes cell proliferation, differentiation, migration, and adhesion. Proliferation and differentiation of stem cells are influenced by the surface of scaffold materials, which have been studied by many researchers in the past decades. Ideal scaffolds for cell survival have the following specific characteristics: firstly, the materials show good biocompatibility; secondly, the materials could be degradable in vivo; thirdly, the fundamental characteristics of materials could mimic the extracellular matrix (ECM) as much as possible [6, 7].

Previous researchers suggested that the scaffold surface microenvironment influenced the fate of stem cells. And the surface microenvironments mainly include physical and biochemical factors [8, 9]. For example, scaffolds with different pore sizes and porosity would lead to different properties and affect the fate of stem cells. Previous studies have shown that scaffolds with pore sizes of 370-400 *μ*m are more conducive to promote the chondrogenic differentiation for ASCs [10, 11]. Also, scaffolds with different materials also affect the fate of stem cells, including cell proliferation, differentiation, and adhesion [12]. It is essential to have a comprehensive understanding of the regulation of the fate of stem cells by physical, biochemical, and other factors, so that we can better design scaffolds with specific microenvironment characteristics to regulate cells for promoting tissue regeneration.

This review summarizes the factors affecting the fate of stem cells which are mainly discussed in terms of physical and chemical aspects: the material stiffness, surface topography, three-dimensional space, mechanical stimulation, and adhesion proteins, growth factors, and substances secreted by cells on the surface of materials. This review is aimed at highlighting the effects of the surface microenvironment of biomaterials in directing stem cell fate.

## 2. Advanced Technology for the Manufacturing of Biomimetic Biomaterials

### 2.1. 3D Printing of Porous Biomimetic Scaffolds

**Figure 1:**
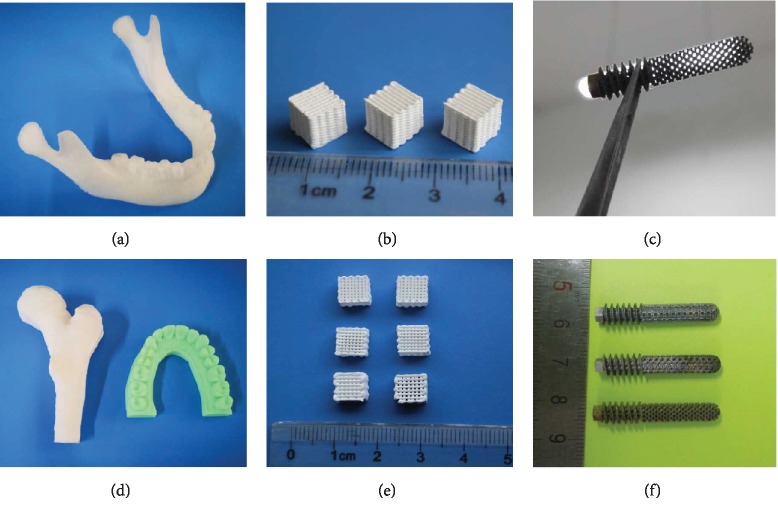
(a, d) Different 3D-printed bone tissue engineering scaffolds. Fused deposition modeling of polymer bone tissue models. (b, e) Direct extrusion 3D printing of calcium phosphate bioceramics. (c, f) Selected laser melting 3D printing of titanium femoral head nail prosthesis [12, 18, 29, 35].

The ideal biomimetic scaffold for tissue reconstruction should resemble natural tissue in both material composition and geometrical properties. For bone tissue biomimetic scaffold, the three-dimensional (3D) porous structure plays a crucial role for bone regeneration [13–16] (Figure 1). This biomimetic porous structure contains interconnected and micro pores and provides a temporary support for cell proliferation and tissue infiltration, as well as a microenvironment for transportation of nutrients and waste products which can function well [17–20]. At the same time, the surface topography of scaffolds also plays an important role in bone tissue regeneration and regulation of cell behaviors. Numerous methods, such as solvent casting/particle leaching [21, 22], phase separation [23, 24], emulsion freeze drying [25], chemical foaming, electrospun, 3D printing, and micropattern techniques [26–29], have been developed to fabricate different porous scaffolds for tissue engineering.

Advances in computational design and 3D printing (3DP) have resulted in quick and accurate fabrication of 3D porous scaffolds with well-controlled geometrical architectures [30–33]. 3DP can fabricate scaffolds with complex internal and external structures in various materials [34–36]. 3DP produces complex scaffolds from a 3D design file by decomposing an object's structures into a series of parallel slices. Internal 3D structures are then fabricated by reproducing these slices one layer at a time by using a sized nozzle (direct extrusion printing) or a programmed selective sintering laser (selective laser melting, SLM), electron beam melting (EBM), or a specific curing light (stereo lithography apparatus, SLA). So far, 3D printing technology has successfully printed various bioceramics, polymers, metal materials, and other biocompatible materials for bone tissue engineering scaffolds [37–39]. These printed scaffolds have highly complicated geometrical architectures with personal-customized shape for different patients in accordance with their CT data. However, the printing capability is limited. For most 3D printing technologies, objects with an accurate porosity of less than 10 *μ*m are difficult to fabricate due to printing accuracy and printing efficiency [40–42].

### 2.2. Electrospinning of Biomimetic Biomaterials

**Figure 2:**
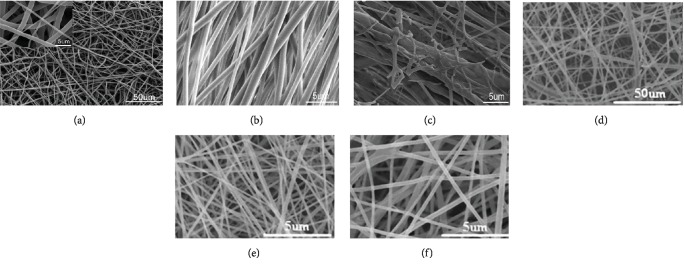
SEM micrographs of different electrospun nanofibers. (a) Electrospun PCL nanofiber [50]. (b) Electrospun-aligned PLGA nanofiber [51]. (c) Electrospun-aligned PLGA/gelatin nanofiber [51]. (d) Electrospun PLA nanofiber [52]. (e) Electrospun silk fibroin-gelatin nanofiber (50 : 50) [52]. (f) Electrospun silk fibroin-gelatin nanofiber (70 : 30) [52].

Electrospinning is curing nanofibers by high-voltage electrostatic force (5-30 kV), which has the advantages of rapid and efficient preparation. In recent years, it has received great attention in the field of tissue engineering. Electrospinning could change the properties by regulating the voltage, conductivity of the solution, distance between the injector and the collector, temperature, and humidity [43]. Common electrospinning materials, including PCL, PLGA, and PLA, have been widely used for tissue regeneration [44–46] (Figure 2). In tendon repairing, orderly arrangement of electrospun nanofibers can guide the arrangement of cells, improve the deposition for ECM, and promote the differentiation of stem cells to regenerate tendon [47]. In addition, electrospun nanofibers could be a suitable carrier, and stem cell could have myogenic differentiation after adding the platelet-derived growth factor (PDGF) [48]. And the arrangement of electrospun nanofibers could be regulated according to requirements. Compared with the random arrangement, the orderly and aligned arrangement of scaffolds showed advantages in neural differentiation of stem cells and migration of neural cell in a rat T9 dorsal hemisection spinal cord injury model, which provided great promise for biomaterial design for applications in nerve regeneration [49].

### 2.3. Micropattern of Biomaterial Surface Topography

**Figure 3:**
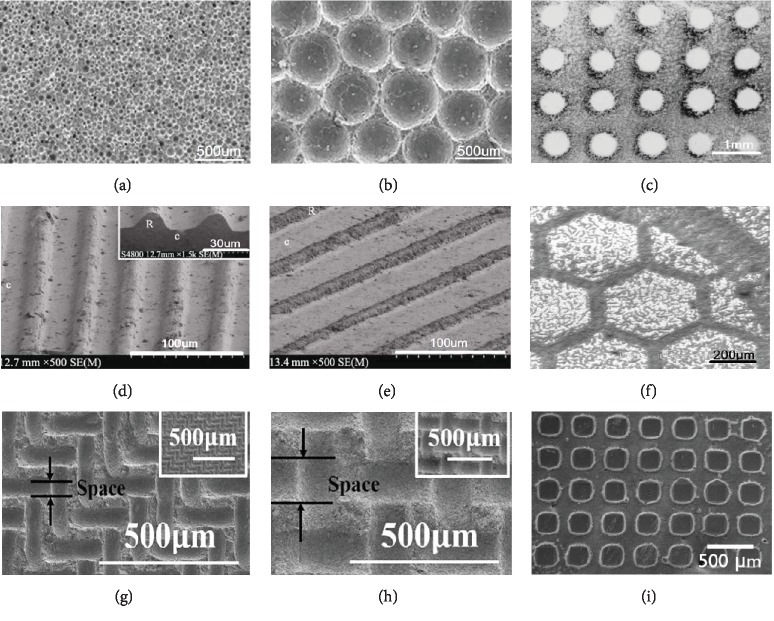
Typical orderly micropatterned scaffold surface. HA bioceramic micropatterned surface with regular small concaves (a) and larger concaves (b) [57]. HA ceramics with spherical array (c) [61]. Micropatterned vertical grooves (d) and inclined grooves (e) [59]. Ordered hexagonal-shape patterns (f) [61]. Quadrate convexes with smaller space (g) and larger space (h) [60]. Grid-shaped patterns (i) [63].

As important factors, the physical and topographical surface of the scaffold could regulate the cell behaviors and control cell function [53, 54]. In addition, a previous study found that the different shapes and sizes of cell could play a role in directing the fates of stem cells [55]. Round cells promoted adipogenesis while cells with high spreading preferred an osteoblast fate by activating MAP kinase pathways and Wnt signaling [53]. In addition, the increased myosin contractility enhances osteogenesis of stem cells. Therefore, the micropatterns of scaffolds could affect the cell behavior by altering the shapes of stem cells [56]. However, these microscopic structures are difficult to fabricate by conventional methods. Literatures reported that the combined uniaxial pressing method and templates may fabricate HA ceramics with regular concaves [57, 58] and grooves [59]. In that work, HA powders were compacted into disc-shaped pellets *via* uniaxial pressing and polystyrene resin microspheres of different sizes were used as poroshifters to form patterned surfaces with a series of regular concaves; the circular holes with diameters of about 50, 200, and 500 *μ*m were patterned uniformly as shown in (Figure 3(a)). *In vitro* studies found that HA bioceramics with 50 *μ*m concaves showed the strongest ability to induce osteogenic differentiation of human osteosarcoma MG-63 cells, as evidenced by the highest alkaline phosphatase (ALP) activity and Cbfa-1 gene expression [57]. Wang et al. reported that HA disc-shaped pellets with micropatterned grooves of ~20, 40, and 60 *μ*m in width were patterned by transferring patterns from different aluminum alloy templates (Figure 3(b)). The HA ceramics with microgrooved patterns showed increased water wettability with decrease of groove width. The microgrooves evidently affected cell elongation, as MC3T3-E1 preosteoblasts were oriented along the direction of grooves, and the cell orientation angles were decreased by decreasing groove width [59]. Zhao et al. [60] fabricated HA ceramics that exhibited micropatterned structured surfaces with quadrate convexes of different sizes *via* uniaxial pressing method by using ordered micropatterned nylon sieves as templates (Figure 3(c)). Compared to the flat one, the micropatterned surface could enhance the adhesion, proliferation, and osteogenic differentiation of rat BMSCs. These studies indicated that bioceramics with regular micropattern of size close to cell size (20-50 *μ*m) showed the best stimulation of cell response.

Furthermore, Wang and Hu [61] created ordered HA patterns with spherical (Figure 3(d)) and hexagonal (Figure 3(e)) shapes on Si and Ti substrates *via* electrophoretic deposition technique. Teshima et al. [62] prepared aligned CaP microstructured patterns with HA nanocrystals by using a hydrophilic/hydrophobic Si-based template photochemically made by VUV light irradiation to provide micro reaction cells for HA crystal growth. Tseng et al. [63] fabricated uniform single-crystal HA nanorods onto specific sites of grid-shaped substrate patterned by hexagonal microcontact printing (Figure 3(f)). However, clear cell behaviors or regulation mechanism of these micropatterned scaffolds remains unidentified, but almost all of the highly ordered patterns close to the diameter of the cells show effective regulation of cell fate.

Surface micropatterning has been widely studied in the preparation of biological functional materials. The patterning methods include photolithography [64], electron beam etching [65], and microcontact transfer method [66, 67]. Traditional methods are usually complicated process and cost high, which limit its application in large-area patterning. The inkjet printing technology is easy to realize direct writing of large-area complex patterns and composite functional materials, which makes it to be a promising method of patterning [68, 69].

## 3. Regulation and Directing of Stem Cell Fate

### 3.1. Scaffold Physical Cues

#### 3.1.1. Pore Size and Porosity Effects

The pore diameter is an essential parameter of the physical structure for porous scaffolds. Pores may determine the nutrition exchange inside of scaffolds, affect the skeletal tension of cell proliferation process, and regulate the fate of stem cells (Table 1). Cells can recognize micropores of 5 nm in the scaffolds. If the pore size is much larger than the cell diameter, the growth situation of the cells will be similar to that on the plate [70]. The pore diameter will affect the adhesion and migration of cells. It is generally believed that scaffolds with a small pore diameter were facilitating the adhesion of cells, while scaffolds with a large pore diameter are more conducive to the migration of cells from the outer layer of scaffolds to the inner layer of scaffolds. In the experiments of osteogenic differentiation of stem cells, it is generally believed that the diameter of 100-300 *μ*m is more conducive to the osteogenic differentiation of bone marrow-derived mesenchymal stem cells [71]. Some scholars have proposed that the pore size of 200 *μ*m is the optimal condition for the osteogenic differentiation of cells [72]. However, 350 *μ*m is considered to be the optimal condition for cell proliferation [73]. When the diameter is larger than 500 *μ*m, cell adhesion will be reduced, which is not conducive to cell proliferation [11]. In terms of cartilage formation, scholars believe that when the diameter is close to 400 *μ*m, it is conducive to cartilage repairing [74]. As for the differentiation of hematopoietic stem cells, it is believed that less than 150 *μ*m is more conducive to the differentiation of stem cells into hematopoietic stem cells [75]. In addition, high porosity could promote the transport of nutrition and oxygen, making it easier for cells to grow inward. However, due to a large number of pores, the mechanical properties of scaffolds will be decreased [76]. The optimal porosity has not been determined, and many studies have shown that scaffolds with high porosity (96.7%) can promote cell proliferation, which may be due to high porosity to promote the transport of nutrients. Some studies showed that when porosity was 86%, cell proliferation was better, which may be because different scaffold materials have different effects on different cells [77].

#### 3.1.2. Stiffness Effects

**Figure 4:**
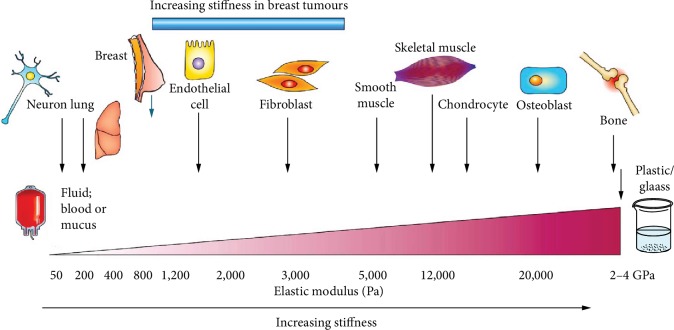
The stiffness affecting the fate of stem cell in vivo, adopted figure from Butcher et al. [98]; the brain is softer than bone, and stem cells are more likely to differentiate into neural differentiation on a soft cell matrix. By contrast, osteogenic differentiation is more likely to occur on scaffolds, which are harder and have material properties similar to those of newly formed bones.

The fate of cells is also affected by the stiffness of the surface microenvironment. Firstly, studies have shown that the stiffness of matrix could affect the differentiation spectrum of stem cell (Figure 4). Stem cells differentiate into muscle cells on soft substrates and osteoblasts on harder substrates [86, 87]. Another study supported this finding, and stem cell on soft materials when stiffness is less than 0.05 kPa could promote neural differentiation effectively, while hard stiffness materials (>40 kPa) promoted osteogenic differentiation effectively [88, 89], which could be related to the Wnt signal pathway [90]. However, there is no agreement on the optimal stiffness for stem cells to differentiate into neurons, muscle cells, cartilage cells, and osteoblasts [86, 91]. Secondly, the stiffness of the material also affects stem cell migration. Stem cells tend to migrate to harder matrix [92]. However, the specific matrix of stem cell migration to the high stiffness matrix is unknown and may be associated with contractility of stem cells [93]. Moreover, the surface stiffness also affects the proliferation of stem cells [94]; a previous study has shown that hydrogels with very soft modulus (~10 Pa) decreased cell proliferation and differentiation [95]. In addition, stiffness is an important factor to maintain the survival rate for stem cells; studies have shown that stem cell on the matrix with a stiffness of 200 Pa survived more than 90% compared to 80% in cultures (100 Pa) [96]. Another study showed that the hardness of 2.5 MPa increased pluripotency [97]. However, the optimal stiffness to maintain pluripotency of stem cells has not been determined, which may be related to different stem cells and material properties from different sources.

#### 3.1.3. Topography Effects

Surface topography plays a vital role in regulating stem cell behavior. In vivo, the topography of the extracellular matrix (ECM) is the basis for cell survival and affects stem cell behavior [99]. In vitro, the surface topography of scaffolds influences the fate of stem cells, including gene expression, cell adhesion, cell proliferation, and extracellular matrix secretion. The scaffold is the cornerstone and directly contacts with stem cell, so the effect of surface topography on stem cells has been widely studied. Surface topography such as roughness and texture is very important in regulating cell response and determining cell fate.

The roughness of the material's surface also plays a role in the fate of stem cells, with a rougher surface reducing the proliferation rate of stem cell compared to a smooth surface. On rough surfaces, cells are more likely to form composite layers, so stem cells are more likely to accumulate in grooves, holes, canyons, and craters, forming bone nodules and ultimately osteogenic differentiation. In the study of Graziano et al., stem cells differentiated faster on concave surfaces and showed nuclear polarity and a high expression of bone-specific proteins, and the interaction between cells and scaffolds is better. However, when cultured on the convex surface, the proliferation activity of stem cells was low, and the extracellular matrix secretion was reduced [100]. Some studies have found that topography can also affect the differentiation lineages of cells. Several lineages including chondrogenic differentiation, osteogenic differentiation, and neuronal differentiation have been studied [101–103].

**Figure 5:**
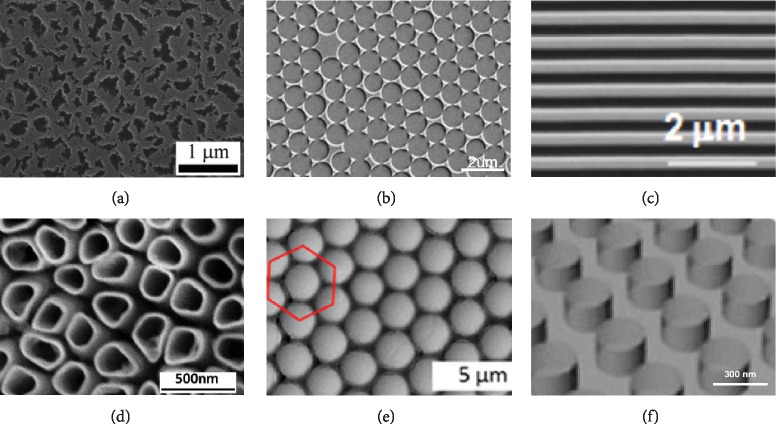
Nanotechnology on different materials with different topographies. (a) Porous silicon fabricated by electrochemical etching, adopted figure from Wang et al. [123]. (b) Colloidal lithography fabricated by self-assembly and sputtering [105]. (c) Nanogrooves fabricated by UV-assisted capillary force lithography [124]. (d) TiO_2_ nanotube fabricated by anodization [125]. (e) Binary colloidal crystals fabricated by self-assembly [126]. (f) Nanopillars (polyurethane acrylate) fabricated by nanoimprinting [127].

In the past decades, the rapid development of nanotechnology has promoted the development of material surface topography modification [104]. Different surface topographies have been reported, such as porous silicon, TiO_2_ nanotube, binary colloidal crystal, colloidal lithography, nanopillars, and nanopillar topographies [105–107] (Figure 5). Nanoscale surface topographies can be constructed by means of electrochemical etching [108, 109], lithography [110, 111], sputtering [112], and colloidal lithography [105, 113, 114]. Each of these methods has advantages and limitations. According to topography forms, nanotech surface topographies could be divided into nanopits, nanocolumns, nanogrooves, and nanotubes. Previous studies have found that ordered nanopits can reduce cell adhesion [115]. However, disordered nanopits can better promote the osteogenic differentiation of embryonic stem cells [116]. Previous studies have found that the height of nanoliths has a great impact on the osteogenic differentiation of stem cells. The height of nanoliths less than 50 nm can stimulate the adhesion of stem cells and improve the osteogenic differentiation, while nanoliths with height of 95 nm were not good for adhesion of stem cells [115]. Nanogrooves are the most common nanoscaffold material, which could promote cell extension or migration, fix cell arrangement, and affect cell differentiation. The arrangement of nanoscale grooves also has an effect on cell fate, and compared with the parallel groove, vertical groove retracted faster [117]. In nanoscale grooves, the ratio of grooves to ridges also influences cell differentiation, and grooves : ridges = 3 : 1 could promote stem cell osteogenesis [118]. In addition, some scholars have discussed the width of groove, and the width of the groove may have an effect on the differentiation spectrum of stem cells, but there is no unified conclusion [116, 119]. As for the limiting sensitivity to grooves, studies have shown that stem cell is sensitive to grooves in 8 nm [120]. However, because of the complexity of manipulating and evaluating cell fate, it is difficult to construct nanoscale materials systematically, and its clinical application is still limited.

In addition, hydrophobicity and chemical moieties are also important factors influencing stem cell behavior. Hydrophilic biomaterial is more conducive to protein adsorption, promoting the transport and excretion of nutrients. Therefore, it is more conducive to tissue regeneration [121, 122]. The chemical composition of the material is similar to that of the host tissue, which is more conducive to the integration of the tissue. For example, calcium phosphate ceramics are chemically similar to natural bone tissue, so they are widely used in bone repair. It was found that this calcium phosphate material could integrate well with bone tissue [16, 37].

#### 3.1.4. Spatial and Dimensional Influences

**Figure 6:**
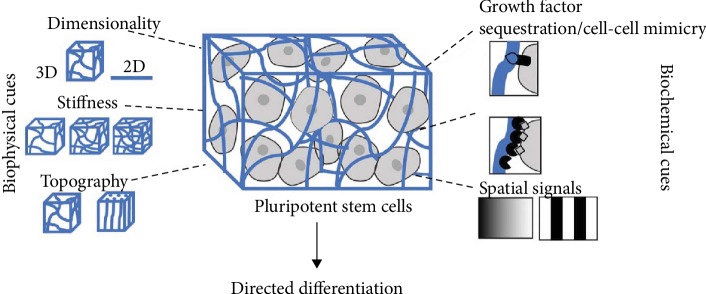
Compared with 2D environment, 3D environment could carry growth factors, maintain stiffness, and promote stem cell differentiation [135].

Cells cultured by a two-dimensional (2D) culture lose their original characteristics in vivo gradually. However, 3D culture could better simulate the living environment of cells in vivo. The cells obtained from a 3D culture were significantly different from those obtained from the 2D culture in terms of morphological structure, proliferation and differentiation, gene expression, and cell function [128]. The 3D cell culture can not only retain the material structure foundation of natural cell microenvironment but also simulate the microenvironment of cell growth *in vivo* (Figure 6), which overcomes the defects of the previous two methods and provides a simpler, safer, and more reliable method for cell research. More and more researches adopt 3D scaffolds for stem cell culture. Some studies have shown that the proliferation and differentiation potential of ASCs is significantly stronger than that in 2D environment when cultured about 21 days [129, 130]. 3D environment prevented the reduction of osteogenic differentiation efficiency of stem cells caused by aging or passage [130]. In the field of tissue engineering, 3D culture could promote the differentiation of stem cells into bone and cartilage compared with 2D culture, which is widely used in the osteochondral tissue engineering [16, 30]. 3D culture also provides a good scaffold for neuron growth, in which neurons could grow in all directions and form a neural network, providing a better method for neuron regeneration [131, 132]. 3D culture can also improve survival of stem cells, as shown in a study by Lee et al., which also found that 3D culture has the advantage of maintaining genomic stability [133]. In the study of Adil et al., 3D culture could generate more neurons with electrophysiological activity, increase cell activity, and integrate well with host tissues after implantation [134].

### 3.2. Scaffold Chemical Cues

#### 3.2.1. Phytochemical Cue Stimulation

The chemical signal of the cell microenvironment can regulate the fate of stem cells. The chemical properties of the surface of the material, such as the characteristics of the material itself, cell coculture, and adhesion between cells could affect the proliferation and differentiation behavior of the cells. For example, many studies have reported that hydroxyapatite itself could promote osteogenic differentiation of stem cells [59]. Some growth factors such as VEGF could promote the differentiation of stem cells into vascular endothelial cells [136]. In our previous study, we have shown that cell coculture could affect the fate of stem cells [137]. Another study showed that coinjection of MSCs and VEGF could affect the fate of stem cell and improve cell implantation myocardial infarction [136, 138].

A large number of studies have been conducted on the effects of phytochemicals on the fate of stem cells. Currently, the phytochemicals studied mainly fall into the following categories: icariin [139], resveratrol [140], quercetin [141], and curcumin [142] (Table 2). Icariin is extracted from the plant herba epimedii and helps improve male fertility [143]. Icariin is associated with phosphorylation of ERK and p38 and activates the ERK and p38 MAPK signaling pathways, leading to the upregulation of MAPK target downstream transcription factors Elk1 and C-MYC, promoting the proliferation of rat BMMSCs. In addition, the optimal concentration of icariin in medium for the proliferation of BMMSCs is 320 *μ*g/L. However, these findings need to be further confirmed in vivo [143]. As a phytoestrogen, resveratrol is a naturally occurring polyphenolic compound in red wine and numerous plants. In addition, resveratrol could activate estrogen receptor signaling selectively. For human mesenchymal stem cells, resveratrol upregulated the expression of osteolineage genes RUNX2 and osteocalcin while suppressing adipolineage genes PPAR*γ*2 and LEPTIN in adipogenic medium, which was mediated mainly through the SIRT1/FOXO3A axis with a smaller contribution from the estrogenic pathway [144]. As an inflammatory demyelinating disease, experimental autoimmune encephalitis is a useful model providing considerable insights into the pathogenesis of multiple sclerosis. The combination of resveratrol and BMMSCs could effectively alleviate the symptoms of autoimmune encephalitis, which is associated with its immunomodulatory effects. The combination of resveratrol and BMMSCs could effectively suppress proinflammatory cytokines (IFN-*γ*, TNF-*α*) and increase anti-inflammatory cytokines (IL-4, IL-10) [145]. Quercetin is one of the most ubiquitous bioflavonoids, widely found in many kinds of plants [141]. Quercetin has a positive pharmacological effect on bone metabolism, which could play a leading role in the quercetin-promoted osteogenic proliferation and differentiation of MSCs by activating the ERK1/2 and JNK signaling pathways [146]. Curcumin is a natural phenolic component of yellow curry spice, which is used in some cultures for the treatment of diseases associated with oxidative stress and inflammation. In addition, curcumin could prevent the death of neurons in animal models of neurodegenerative disorders [142]. Kim et al. conducted a research to investigate the effects of curcumin on mouse multipotent neural progenitor cells and adult hippocampal neurogenesis. The results showed that curcumin could promote the proliferation and neural differentiation of hippocampal embryonic stem cells at low concentrations and be cytotoxic at high concentrations. In addition, curcumin could activate cellular signal transduction pathways, including ERK and p38MAPK pathways, which could regulate neuronal plasticity and stress responses [147]. In conclusion, phytochemical stimulation regulates the fate of stem cells by regulating signal pathways such as Wnt, protein kinase, and PI3K/Akt signaling pathways.

#### 3.2.2. Cell-Adhesive Ligand Effects

**Figure 7:**
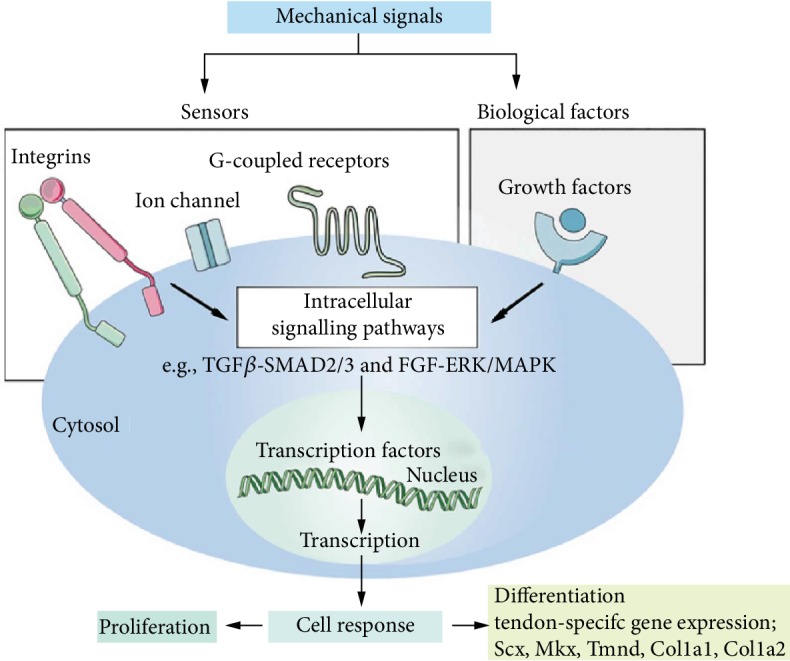
Mechanical signal transduction. Mechanical signaling influences the proliferation and differentiation of stem cells through integrins, ion channels, receptors or exogenous growth factors, and complex intracellular pathways [171].

The adhesion of cells and their surroundings is very important to the fate of stem cells, which can regulate the apoptosis, migration, and differentiation of stem cells [158]. This cellular adhesion to the microenvironment is mediated by transmembrane matrix receptors (Figure 7). Integrin is an important transmembrane receptor that plays an important role in signal transduction by mediating the main link between cells and ECM [159]. Integrin is a heterodimer transmembrane molecule composed of different alpha and beta subunits that binds directly to ECM proteins such as collagen, laminin, and fibronectin. Integrins bind to adhesion molecules (CD54 or ICAM1) on the cell surface and adhesion molecules (CD106 or VCAM1) which are present in stem cells. However, in in vitro culture, the expression of integrin is different due to different cell sources and culture methods. RGD is an integrin-binding ligand, which could be used to explore the interaction between cells and ECM [160]. Studies have shown that changing the coupling strength of RGD peptide on substrates could regulate the adhesion, diffusion, and differentiation of MSCs [161]. By adding RGD-related polypeptide into hydrogel, cell adhesion and diffusion could be promoted while high concentrations of RGD also inhibit cell detachment [162]. Due to the importance of adhesion between cells and matrix, strategies for adding binding ligands to hydrogels have been studied. Luo et al. discovered an agarose hydrogel which could react with RGD peptides by exposure to light [163]. In addition to RGD, other adhesion peptides, such as YIGSR and IKVAV, could also influence the fate of stem cell [164, 165]. Integrin, adaptor, and signal proteins together form the adhesive plaque complex, which contains more than 100 proteins which connect actomyosin and ECM and form the signaling pathway [166, 167]. In addition to integrins, cadherins are important receptors on cell surfaces and are involved in stem cell migration and homing [168]. Cadherins play an important role in stem cell early adhesion and self-renewal [169]. The study of cadherins is limited now, and more researchers are needed in the future. In addition to integrin and cadherin, other cell surface receptors are also considered important for stem-niche interactions, including EGF, Notch, curl, TGF beta, gap junction, c-kit, CD44, and VCAM1 [170].

#### 3.2.3. Growth Factor Effects

The development and differentiation of stem cells are affected by various internal mechanisms and microenvironmental factors, and growth factors are often used as inducers of differentiation (Figure 7). Therefore, it is very important to clarify their role in the survival or differentiation of stem cells. There are also growth factors that mobilize stem cells to return home for tissue repair. The most common growth factor includes platelet-derived growth factor, insulin-like growth factor-1, hepatocyte growth factor (HGF), EGF, and angiopoietin [172–176]. Currently, growth factor is widely used in the field of regeneration, such as bone regeneration and cartilage regeneration. There are many cytokines that promote bone formation, such as BMP, PDGF, TGF-beta, FGF, and IGF [177]. Among them, BMP is the most widely used osteogenic factor. BMP could induce MSC proliferation and differentiation into chondrocytes and osteoblasts [178]. In terms of heart repair, literatures reported that coinjection of MSCs and VEGF into the heart with myocardial infarction increased cell implantation and resulted in better cardiac function than either VEGF or MSC alone [136, 179]. Mesenchymal stem cells with IGF-1 overexpression promote bone marrow stem cell mobilization through paracrine activation of SDF-1alpha/CXCR4 signaling so as to promote cardiac repair [138]. The combination of laminin and platelet-derived growth factor could promote neuronal differentiation of U-MSCs [180]. Hepatocyte growth factor could promote the differentiation of stem cells, which may be associated with the activation of Wnt signaling [181]. Another study found that this hepatocyte growth factor significantly promotes the viability of embryo-derived mesenchymal stem cells and prevents its senescence, which is associated with transcription of RAD51 [182]. All of the above growth factors have an impact on the proliferation and differentiation of stem cells. Loading growth factors onto the scaffold material could affect the growth of stem cells, which could be the direction of tissue engineering research. Local sustained release is an important part of how to use growth factors efficiently.

## 4. Conclusion and Future Perspective

The fate of stem cells in the body is complicated and much remains unknown. The fate of stem cells is regulated not only by the genetic material but also by the microenvironment. The ideal microenvironment is a combination of various conditions to simulate the extracellular matrix as much as possible, to construct the physicochemical conditions suitable for the growth of stem cells, and to meet the requirements of proliferation, differentiation, adhesion, and other aspects of stem cells. Ideal microenvironments include a proper mechanical stiffness, porosity, aperture, topography, 3D environment, proper mechanical stimulation, and orderly/disordered arrangement. It is generally believed that scaffolds with a pore diameter of 100-300 *μ*m are more conducive to the osteogenic differentiation of bone marrow-derived mesenchymal stem cells. When the diameter is larger than 500 *μ*m, cell adhesion will be reduced, which is not conducive to cell proliferation. In terms of cartilage formation, it is generally accepted that when the diameter is close to 400 *μ*m, it is conducive to cartilage repairing. Stiffness is an important factor to maintain the survival rate for stem cells. Stem cells tend to migrate to harder matrix. Different substrates with varied stiffness would affect stem cell differentiation. In addition, exogenous phytochemicals, peptides, and growth factors will stimulate stem cells through a series of complex signaling pathways, affecting the fate of stem cells. Changing the microenvironment to guide stem cell behavior is challenging because of the complex structure of cells and some unknown signaling pathways, which require greater efforts in the future. With the development of fabrication techniques, there are many advance fabrication methods, such as 3D printing, electrospinning, and micropatterning, which were successfully applied to design and fabricate scaffolds with specific microenvironment [183].

At present, many researchers have promoted stem cell differentiation and tissue regeneration by adding growth factors. However, studies have shown that matrix characteristics may be more important than exogenous addition of growth or differentiation factors, which may provide a direction for future research [86]. This review highlights the contribution of physical and chemical cues that influence stem cell fate. Most of the current studies are preclinical, and their progress in clinical applications requires additional testing to demonstrate safety and efficacy. In addition, it was found that the same materials have different effects on the fate of stem cells from different sources. Proper stem cell and matched surface microenvironment remain the focus of future research. By combining these strategies with existing material properties to guide cell fate, stem cells could be an important option in tissue engineering. Although there are many factors and cues that can regulate the release of growth factors, they have advantages and disadvantages and need to be selected according to the specific situation.

## Figures and Tables

**Table 1:** Proposed optimal pore sizes and porosities affecting the fate of stem cell.

Material	Optimal pore size (*μ*m)	Optimal porosity (%)	Target stem cell(s)	Potential application(s)	Reference
*β*-Tricalcium phosphate	200-600	65	BMMSCs	Osteogenic	[78]
Sintered titanium fiber mesh	250	86	BMMSCs	Osteogenic	[79]
PCL	200		ASCs	Proliferation	[74]
PCL	400		ASCs	Chondrogenic	[74]
Polycaprolactone	370–400	80–97	BMMSCs	Chondrogenic	[11]
Poly(lactic-*co*-glycolic acid)	120–200	50	ASCs	Hepatogenesis	[80]
Poly(lactic-*co*-glycolic acid)	50–200		BMMSCs	Myogenic	[81]
Coralline hydroxyapatite	200	75	BMMSCs	Osteogenic	[82]
*β*-Tricalcium phosphate	400–500	70	BMMSCs	Osteogenic	[83]
ZrO_2_ ceramic	600	80–89	ASCs	Osteogenic	[84]
Polycaprolactone	100–150		BMMSCs	Chondrogenic	[85]

**Table 2:** The applications of phytochemicals for stem cell.

Phytochemical	Affecting signal transduction pathway	Target stem cell(s)	Potential application(s)	Reference
Icariin	PI3K/Akt and STAT3	ASCs	Diabetes-associated erectile dysfunction	[148]
	ERK and p38 MAPK	BMMSCs	Proliferation	[143]
	SDF-1alpha/HIF-1alpha/CXCR4	BMMSCs	Migration	[139]
	PI3K and ERK1/2	BMMSCs	Angiogenesis and neurogenesis	[149]
Resveratrol	SIRT1/FOXO3A	Human embryonic stem cells	Osteoblastic differentiation	[144]
	AMPK	BMMSCs	Osteogenic differentiation	[140]
	AMPK/Ulk1	Embryonic stem cells	Pluripotency	[150]
	SIRT1	Umbilical cord-derived mesenchymal stem cells	Neural repair of Alzheimer's disease	[151]
Quercetin	p38 MAPK, ERK1/2, and JNK	BMMSCs	Osteogenesis	[141]
	TNF-alpha	BMMSCs	Osteogenesis	[152]
	BMP2, Smad1, Smad4, RUNX2, OSX, and OPN expression and Smad1 phosphorylation	BMMSCs	Differentiation	[153]
Curcumin	Self-renewal genes, Notch1 and Hes1	Neural stem cells	Proliferation	[154]
	Caveolin-1	Epidermal stem cells	Proliferation	[155]
	Glucocorticoid receptor and STAT3	Embryonic neural stem cells	Proliferation	[156]
	TERT gene	ASCs	Improve lifespan	[157]
